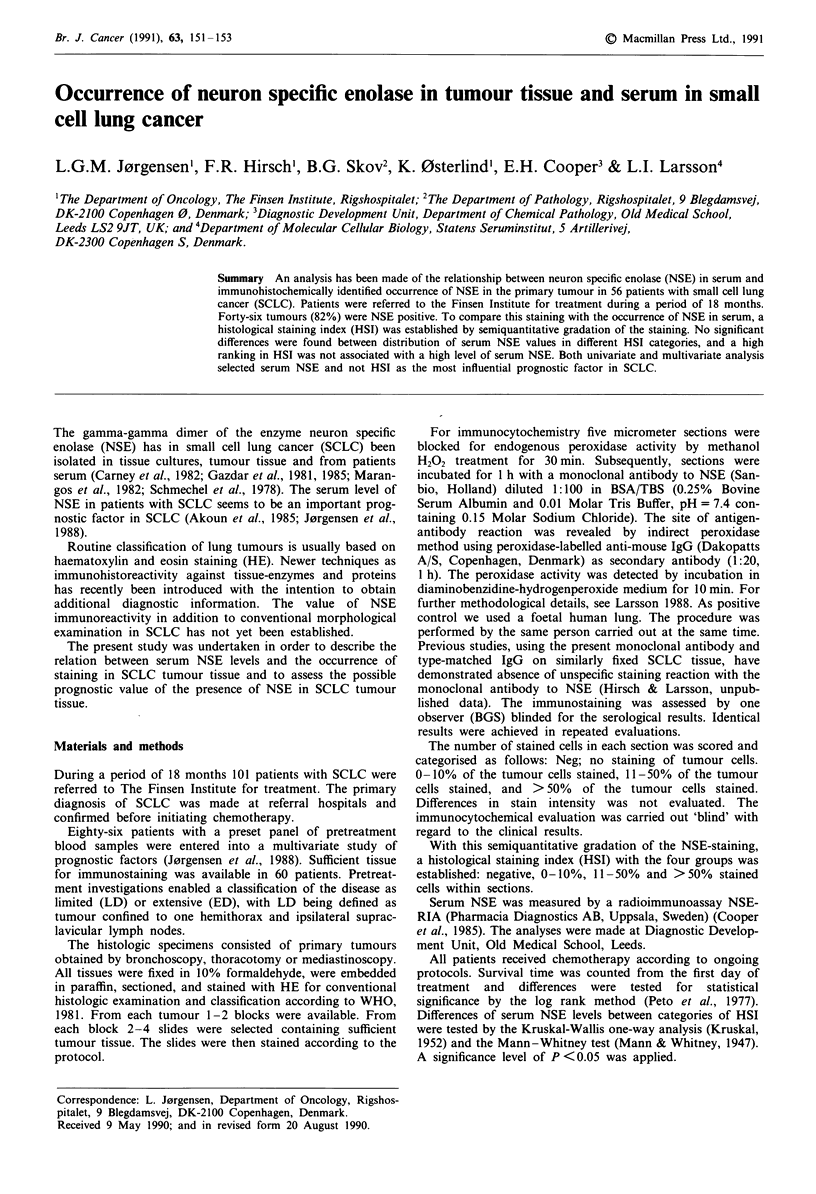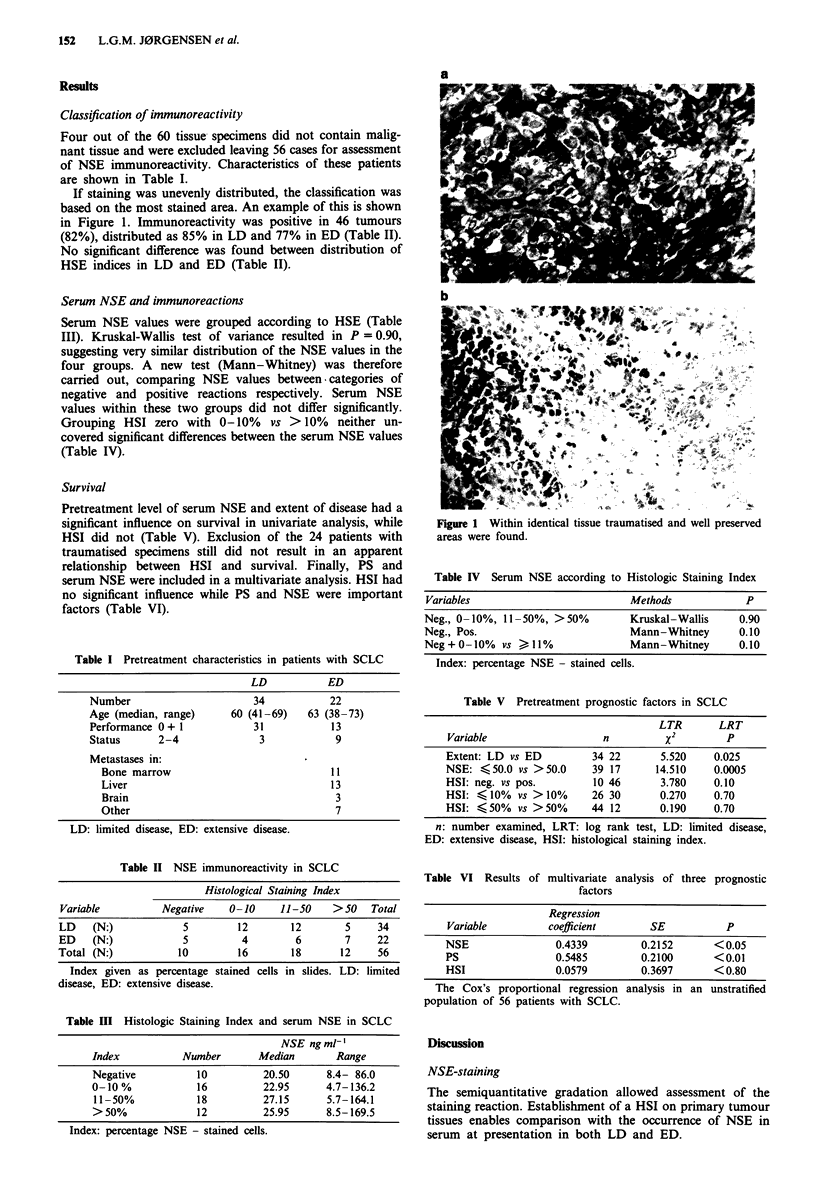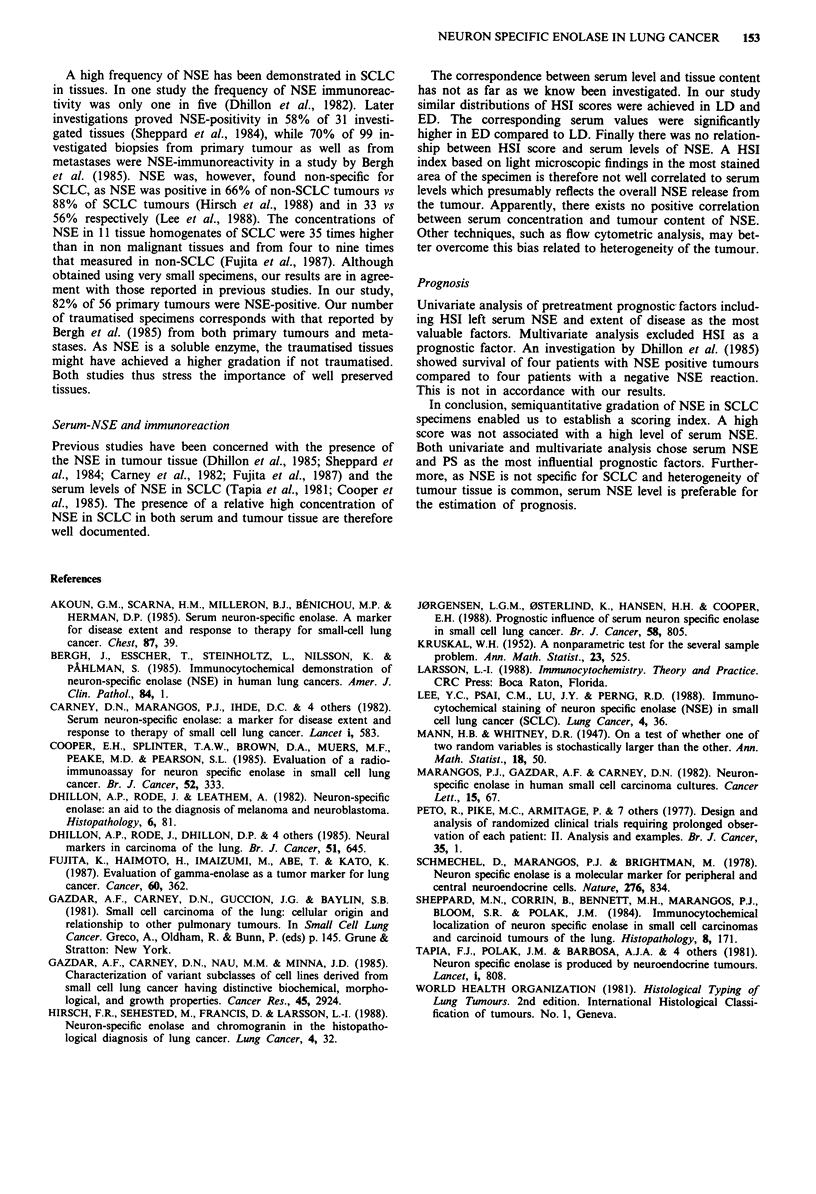# Occurrence of neuron specific enolase in tumour tissue and serum in small cell lung cancer.

**DOI:** 10.1038/bjc.1991.31

**Published:** 1991-01

**Authors:** L. G. Jørgensen, F. R. Hirsch, B. G. Skov, K. Osterlind, E. H. Cooper, L. I. Larsson

**Affiliations:** Department of Oncology, Finsen Institute, Rigshospitalet, Copenhagen, Denmark.

## Abstract

**Images:**


Br. J. Cancer (1991), 63, 151-153 © Macmillan Press Ltd., 1991

### Occurrence of neuron specific enolase in tumour tissue and serum in small cell lung cancer

L.G.M. Jørgensen[1], F.R. Hirsch[1], B.G. Skov[2], K. Østerlind[1], E.H. Cooper[3] & L.I. Larsson[4]

[1]*The Department of Oncology, The Finsen Institute, Rigshospitalet;* [2]*The Department of Pathology, Rigshospitalet, 9 Blegdamsvej, DK-2100 Copenhagen Ø, Denmark;* [3]*Diagnostic Development Unit, Department of Chemical Pathology, Old Medical School, Leeds LS2 9JT, UK; and* [4]*Department of Molecular Cellular Biology, Statens Seruminstitut, 5 Artillerivej, DK-2300 Copenhagen S, Denmark.*

**Summary** An analysis has been made of the relationship between neuron specific enolase (NSE) in serum and immunohistochemically identified occurrence of NSE in the primary tumour in 56 patients with small cell lung cancer (SCLC). Patients were referred to the Finsen Institute for treatment during a period of 18 months. Forty-six tumours (82%) were NSE positive. To compare this staining with the occurrence of NSE in serum, a histological staining index (HSI) was established by semiquantitative gradation of the staining. No significant differences were found between distribution of serum NSE values in different HSI categories, and a high ranking in HSI was not associated with a high level of serum NSE. Both univariate and multivariate analysis selected serum NSE and not HSI as the most influential prognostic factor in SCLC.

The gamma-gamma dimer of the enzyme neuron specific enolase (NSE) has in small cell lung cancer (SCLC) been isolated in tissue cultures, tumour tissue and from patients serum (Carney *et al.*, 1982; Gazdar *et al.*, 1981, 1985; Marangos *et al.*, 1982; Schmechel *et al.*, 1978). The serum level of NSE in patients with SCLC seems to be an important prognostic factor in SCLC (Akoun *et al.*, 1985; Jørgensen *et al.*, 1988).

Routine classification of lung tumours is usually based on haematoxylin and eosin staining (HE). Newer techniques as immunohistoreactivity against tissue-enzymes and proteins has recently been introduced with the intention to obtain additional diagnostic information. The value of NSE immunoreactivity in addition to conventional morphological examination in SCLC has not yet been established.

The present study was undertaken in order to describe the relation between serum NSE levels and the occurrence of staining in SCLC tumour tissue and to assess the possible prognostic value of the presence of NSE in SCLC tumour tissue.

### Materials and methods

During a period of 18 months 101 patients with SCLC were referred to The Finsen Institute for treatment. The primary diagnosis of SCLC was made at referral hospitals and confirmed before initiating chemotherapy.

Eighty-six patients with a preset panel of pretreatment blood samples were entered into a multivariate study of prognostic factors (Jørgensen *et al.*, 1988). Sufficient tissue for immunostaining was available in 60 patients. Pretreatment investigations enabled a classification of the disease as limited (LD) or extensive (ED), with LD being defined as tumour confined to one hemithorax and ipsilateral supraclavicular lymph nodes.

The histologic specimens consisted of primary tumours obtained by bronchoscopy, thoracotomy or mediastinoscopy. All tissues were fixed in 10% formaldehyde, were embedded in paraffin, sectioned, and stained with HE for conventional histologic examination and classification according to WHO, 1981. From each tumour 1-2 blocks were available. From each block 2-4 slides were selected containing sufficient tumour tissue. The slides were then stained according to the protocol.

For immunocytochemistry five micrometer sections were blocked for endogenous peroxidase activity by methanol $H_2O_2$ treatment for 30 min. Subsequently, sections were incubated for 1 h with a monoclonal antibody to NSE (Sanbio, Holland) diluted 1:100 in BSA/TBS (0.25% Bovine Serum Albumin and 0.01 Molar Tris Buffer, pH = 7.4 containing 0.15 Molar Sodium Chloride). The site of antigen-antibody reaction was revealed by indirect peroxidase method using peroxidase-labelled anti-mouse IgG (Dakopatts A/S, Copenhagen, Denmark) as secondary antibody (1:20, 1 h). The peroxidase activity was detected by incubation in diaminobenzidine-hydrogenperoxide medium for 10 min. For further methodological details, see Larsson 1988. As positive control we used a foetal human lung. The procedure was performed by the same person carried out at the same time. Previous studies, using the present monoclonal antibody and type-matched IgG on similarly fixed SCLC tissue, have demonstrated absence of unspecific staining reaction with the monoclonal antibody to NSE (Hirsch & Larsson, unpublished data). The immunostaining was assessed by one observer (BGS) blinded for the serological results. Identical results were achieved in repeated evaluations.

The number of stained cells in each section was scored and categorised as follows: Neg; no staining of tumour cells. 0-10% of the tumour cells stained, 11-50% of the tumour cells stained, and >50% of the tumour cells stained. Differences in stain intensity was not evaluated. The immunocytochemical evaluation was carried out 'blind' with regard to the clinical results.

With this semiquantitative gradation of the NSE-staining, a histological staining index (HSI) with the four groups was established: negative, 0-10%, 11-50% and >50% stained cells within sections.

Serum NSE was measured by a radioimmunoassay NSE-RIA (Pharmacia Diagnostics AB, Uppsala, Sweden) (Cooper *et al.*, 1985). The analyses were made at Diagnostic Development Unit, Old Medical School, Leeds.

All patients received chemotherapy according to ongoing protocols. Survival time was counted from the first day of treatment and differences were tested for statistical significance by the log rank method (Peto *et al.*, 1977). Differences of serum NSE levels between categories of HSI were tested by the Kruskal-Wallis one-way analysis (Kruskal, 1952) and the Mann-Whitney test (Mann & Whitney, 1947). A significance level of $P < 0.05$ was applied.

Correspondence: L. Jørgensen, Department of Oncology, Rigshospitalet, 9 Blegdamsvej, DK-2100 Copenhagen, Denmark.
Received 9 May 1990; and in revised form 20 August 1990.

### Results

### Classification of immunoreactivity

Four out of the 60 tissue specimens did not contain malignant tissue and were excluded leaving 56 cases for assessment of NSE immunoreactivity. Characteristics of these patients are shown in Table I.

If staining was unevenly distributed, the classification was based on the most stained area. An example of this is shown in Figure 1. Immunoreactivity was positive in 46 tumours (82%), distributed as 85% in LD and 77% in ED (Table II). No significant difference was found between distribution of HSE indices in LD and ED (Table II).

### Serum NSE and immunoreactions

Serum NSE values were grouped according to HSE (Table III). Kruskal-Wallis test of variance resulted in $P = 0.90$, suggesting very similar distribution of the NSE values in the four groups. A new test (Mann–Whitney) was therefore carried out, comparing NSE values between categories of negative and positive reactions respectively. Serum NSE values within these two groups did not differ significantly. Grouping HSI zero with 0–10% *vs* >10% neither uncovered significant differences between the serum NSE values (Table IV).

### Survival

Pretreatment level of serum NSE and extent of disease had a significant influence on survival in univariate analysis, while HSI did not (Table V). Exclusion of the 24 patients with traumatised specimens still did not result in an apparent relationship between HSI and survival. Finally, PS and serum NSE were included in a multivariate analysis. HSI had no significant influence while PS and NSE were important factors (Table VI).

**Table I** Pretreatment characteristics in patients with SCLC

| | *LD* | *ED* |
|---|---|---|
| Number | 34 | 22 |
| Age (median, range) | 60 (41–69) | 63 (38–73) |
| Performance 0 + 1 | 31 | 13 |
| Status 2–4 | 3 | 9 |
| Metastases in: | | |
| Bone marrow | | 11 |
| Liver | | 13 |
| Brain | | 3 |
| Other | | 7 |

LD: limited disease, ED: extensive disease.

**Table II** NSE immunoreactivity in SCLC

| | *Histological Staining Index* | | | | |
|---|---|---|---|---|---|
| *Variable* | *Negative* | *0–10* | *11–50* | *>50* | *Total* |
| LD (N:) | 5 | 12 | 12 | 5 | 34 |
| ED (N:) | 5 | 4 | 6 | 7 | 22 |
| Total (N:) | 10 | 16 | 18 | 12 | 56 |

Index given as percentage stained cells in slides. LD: limited disease, ED: extensive disease.

**Table III** Histologic Staining Index and serum NSE in SCLC

| | | *NSE ng ml⁻¹* | |
|---|---|---|---|
| *Index* | *Number* | *Median* | *Range* |
| Negative | 10 | 20.50 | 8.4– 86.0 |
| 0–10 % | 16 | 22.95 | 4.7–136.2 |
| 11–50% | 18 | 27.15 | 5.7–164.1 |
| >50% | 12 | 25.95 | 8.5–169.5 |

Index: percentage NSE – stained cells.

**Figure 1** Within identical tissue traumatised and well preserved areas were found.

**Table IV** Serum NSE according to Histologic Staining Index

| *Variables* | *Methods* | *P* |
|---|---|---|
| Neg., 0–10%, 11–50%, >50% | Kruskal–Wallis | 0.90 |
| Neg., Pos. | Mann–Whitney | 0.10 |
| Neg + 0–10% *vs* ≥11% | Mann–Whitney | 0.10 |

Index: percentage NSE – stained cells.

**Table V** Pretreatment prognostic factors in SCLC

| *Variable* | *n* | *LTR* $\chi^2$ | *LRT* *P* |
|---|---|---|---|
| Extent: LD *vs* ED | 34 22 | 5.520 | 0.025 |
| NSE: ≤50.0 *vs* >50.0 | 39 17 | 14.510 | 0.0005 |
| HSI: neg. *vs* pos. | 10 46 | 3.780 | 0.10 |
| HSI: ≤10% *vs* >10% | 26 30 | 0.270 | 0.70 |
| HSI: ≤50% *vs* >50% | 44 12 | 0.190 | 0.70 |

*n*: number examined, LRT: log rank test, LD: limited disease, ED: extensive disease, HSI: histological staining index.

**Table VI** Results of multivariate analysis of three prognostic factors

| *Variable* | *Regression coefficient* | *SE* | *P* |
|---|---|---|---|
| NSE | 0.4339 | 0.2152 | <0.05 |
| PS | 0.5485 | 0.2100 | <0.01 |
| HSI | 0.0579 | 0.3697 | <0.80 |

The Cox's proportional regression analysis in an unstratified population of 56 patients with SCLC.

### Discussion

### NSE-staining

The semiquantitative gradation allowed assessment of the staining reaction. Establishment of a HSI on primary tumour tissues enables comparison with the occurrence of NSE in serum at presentation in both LD and ED.

A high frequency of NSE has been demonstrated in SCLC in tissues. In one study the frequency of NSE immunoreactivity was only one in five (Dhillon *et al.*, 1982). Later investigations proved NSE-positivity in 58% of 31 investigated tissues (Sheppard *et al.*, 1984), while 70% of 99 investigated biopsies from primary tumour as well as from metastases were NSE-immunoreactivity in a study by Bergh *et al.* (1985). NSE was, however, found non-specific for SCLC, as NSE was positive in 66% of non-SCLC tumours *vs* 88% of SCLC tumours (Hirsch *et al.*, 1988) and in 33 *vs* 56% respectively (Lee *et al.*, 1988). The concentrations of NSE in 11 tissue homogenates of SCLC were 35 times higher than in non malignant tissues and from four to nine times that measured in non-SCLC (Fujita *et al.*, 1987). Although obtained using very small specimens, our results are in agreement with those reported in previous studies. In our study, 82% of 56 primary tumours were NSE-positive. Our number of traumatised specimens corresponds with that reported by Bergh *et al.* (1985) from both primary tumours and metastases. As NSE is a soluble enzyme, the traumatised tissues might have achieved a higher gradation if not traumatised. Both studies thus stress the importance of well preserved tissues.

*Serum-NSE and immunoreaction*

Previous studies have been concerned with the presence of the NSE in tumour tissue (Dhillon *et al.*, 1985; Sheppard *et al.*, 1984; Carney *et al.*, 1982; Fujita *et al.*, 1987) and the serum levels of NSE in SCLC (Tapia *et al.*, 1981; Cooper *et al.*, 1985). The presence of a relative high concentration of NSE in SCLC in both serum and tumour tissue are therefore well documented.

The correspondence between serum level and tissue content has not as far as we know been investigated. In our study similar distributions of HSI scores were achieved in LD and ED. The corresponding serum values were significantly higher in ED compared to LD. Finally there was no relationship between HSI score and serum levels of NSE. A HSI index based on light microscopic findings in the most stained area of the specimen is therefore not well correlated to serum levels which presumably reflects the overall NSE release from the tumour. Apparently, there exists no positive correlation between serum concentration and tumour content of NSE. Other techniques, such as flow cytometric analysis, may better overcome this bias related to heterogeneity of the tumour.

*Prognosis*

Univariate analysis of pretreatment prognostic factors including HSI left serum NSE and extent of disease as the most valuable factors. Multivariate analysis excluded HSI as a prognostic factor. An investigation by Dhillon *et al.* (1985) showed survival of four patients with NSE positive tumours compared to four patients with a negative NSE reaction. This is not in accordance with our results.

In conclusion, semiquantitative gradation of NSE in SCLC specimens enabled us to establish a scoring index. A high score was not associated with a high level of serum NSE. Both univariate and multivariate analysis chose serum NSE and PS as the most influential prognostic factors. Furthermore, as NSE is not specific for SCLC and heterogeneity of tumour tissue is common, serum NSE level is preferable for the estimation of prognosis.

**References**

AKOUN, G.M., SCARNA, H.M., MILLERON, B.J., BÉNICHOU, M.P. & HERMAN, D.P. (1985). Serum neuron-specific enolase. A marker for disease extent and response to therapy for small-cell lung cancer. *Chest*, **87**, 39.

BERGH, J., ESSCHER, T., STEINHOLTZ, L., NILSSON, K. & PÅHLMAN, S. (1985). Immunocytochemical demonstration of neuron-specific enolase (NSE) in human lung cancers. *Amer. J. Clin. Pathol.*, **84**, 1.

CARNEY, D.N., MARANGOS, P.J., IHDE, D.C. & 4 others (1982). Serum neuron-specific enolase: a marker for disease extent and response to therapy of small cell lung cancer. *Lancet* **i**, 583.

COOPER, E.H., SPLINTER, T.A.W., BROWN, D.A., MUERS, M.F., PEAKE, M.D. & PEARSON, S.L. (1985). Evaluation of a radioimmunoassay for neuron specific enolase in small cell lung cancer. *Br. J. Cancer*, **52**, 333.

DHILLON, A.P., RODE, J. & LEATHEM, A. (1982). Neuron-specific enolase: an aid to the diagnosis of melanoma and neuroblastoma. *Histopathology*, **6**, 81.

DHILLON, A.P., RODE, J., DHILLON, D.P. & 4 others (1985). Neural markers in carcinoma of the lung. *Br. J. Cancer*, **51**, 645.

FUJITA, K., HAIMOTO, H., IMAIZUMI, M., ABE, T. & KATO, K. (1987). Evaluation of gamma-enolase as a tumor marker for lung cancer. *Cancer*, **60**, 362.

GAZDAR, A.F., CARNEY, D.N., GUCCION, J.G. & BAYLIN, S.B. (1981). Small cell carcinoma of the lung: cellular origin and relationship to other pulmonary tumours. In *Small Cell Lung Cancer*. Greco, A., Oldham, R. & Bunn, P. (eds) p. 145. Grune & Stratton: New York.

GAZDAR, A.F., CARNEY, D.N., NAU, M.M. & MINNA, J.D. (1985). Characterization of variant subclasses of cell lines derived from small cell lung cancer having distinctive biochemical, morphological, and growth properties. *Cancer Res.*, **45**, 2924.

HIRSCH, F.R., SEHESTED, M., FRANCIS, D. & LARSSON, L.-I. (1988). Neuron-specific enolase and chromogranin in the histopathological diagnosis of lung cancer. *Lung Cancer*, **4**, 32.

JØRGENSEN, L.G.M., ØSTERLIND, K., HANSEN, H.H. & COOPER, E.H. (1988). Prognostic influence of serum neuron specific enolase in small cell lung cancer. *Br. J. Cancer*, **58**, 805.

KRUSKAL, W.H. (1952). A nonparametric test for the several sample problem. *Ann. Math. Statist.*, **23**, 525.

LARSSON, L.-I. (1988). *Immunocytochemistry. Theory and Practice*. CRC Press: Boca Raton, Florida.

LEE, Y.C., PSAI, C.M., LU, J.Y. & PERNG, R.D. (1988). Immunocytochemical staining of neuron specific enolase (NSE) in small cell lung cancer (SCLC). *Lung Cancer*, **4**, 36.

MANN, H.B. & WHITNEY, D.R. (1947). On a test of whether one of two random variables is stochastically larger than the other. *Ann. Math. Statist.*, **18**, 50.

MARANGOS, P.J., GAZDAR, A.F. & CARNEY, D.N. (1982). Neuron-specific enolase in human small cell carcinoma cultures. *Cancer Lett.*, **15**, 67.

PETO, R., PIKE, M.C., ARMITAGE, P. & 7 others (1977). Design and analysis of randomized clinical trials requiring prolonged observation of each patient: II. Analysis and examples. *Br. J. Cancer*, **35**, 1.

SCHMECHEL, D., MARANGOS, P.J. & BRIGHTMAN, M. (1978). Neuron specific enolase is a molecular marker for peripheral and central neuroendocrine cells. *Nature*, **276**, 834.

SHEPPARD, M.N., CORRIN, B., BENNETT, M.H., MARANGOS, P.J., BLOOM, S.R. & POLAK, J.M. (1984). Immunocytochemical localization of neuron specific enolase in small cell carcinomas and carcinoid tumours of the lung. *Histopathology*, **8**, 171.

TAPIA, F.J., POLAK, J.M. & BARBOSA, A.J.A. & 4 others (1981). Neuron specific enolase is produced by neuroendocrine tumours. *Lancet*, **i**, 808.

WORLD HEALTH ORGANIZATION (1981). *Histological Typing of Lung Tumours*. 2nd edition. International Histological Classification of tumours. No. 1, Geneva.